# Macronutrient deficiency in snap bean considering physiological, nutritional, and growth aspects

**DOI:** 10.1371/journal.pone.0234512

**Published:** 2020-06-08

**Authors:** Christian Rones Wruck de Souza Osório, Gelza Carliane Marques Teixeira, Rafael Ferreira Barreto, Cid Naudi Silva Campos, Aguinaldo José Freitas Leal, Paulo Eduardo Teodoro, Renato de Mello Prado

**Affiliations:** 1 Federal University of Mato Grosso do Sul (UFMS), Chapadão do Sul, MS, Brazil; 2 Department of Soils and Fertilizers, Laboratory of Plant Nutrition, São Paulo State University (UNESP), Jaboticabal, SP, Brazil; 3 Federal University of Triângulo Mineiro (UFTM), Iturama, MG, Brazil; United Arab Emirates University, UNITED ARAB EMIRATES

## Abstract

Nutritional deficiencies limit the growth of snap bean plants, therefore knowing the biological mechanisms involved in it is fundamental. This study is aimed to evaluate the damage caused by a deficiency of macronutrients in physiological variables that cause decreased growth and the appearance of visual symptoms in snap bean. Thus, we design a hydroponic system of snap bean cultivation in order to test the effect of macronutrient deficiencies in a controlled environment. The treatments consisted in evaluate the effects of lack of one macronutrient in time. To perform this, we used Hoagland and Arnon solution in its complete formulation (control) or without N, P; K; Mg, Ca or S in each treatment. Physiological, nutritional, and growth analyses were performed when visual deficiency symptoms of each omitted nutrient appeared. Thus, the omissions of N and P in the nutrient solution led to lower accumulations of all macronutrients in the shoot. And the K, Ca, Mg, and S omissions decreased the amounts of K, Ca, Mg, P, and S in the shoot of the snap bean plants when compared with the plants grown in the complete nutrient solution. With the lowest accumulation of macronutrients, the content of photosynthetic pigments and the photosynthetic rate were reduced, with harmful effects on plant growth. Thus, from the losses in dry matter production of the shoot, the order of limiting of macronutrients in bean plants was N < P < Ca < S < Mg < K, with a decrease of up to 86.2%, 80.1%, 51.2%, 46.5%, 25.6%, and 19.3%, respectively. The nitrogen deficiency is more evident, proven by symptoms such as chlorosis in the lower and upper third leaves and necrosis of the lower third leaves.

## Introduction

Snap bean (*Phaseolus vulgaris* L.) belongs to the Fabaceae family, and its cultivation is destined to the production of edible pods, with high market demand [1]. It has an indeterminate growth habit, with a production cycle composed of periodic harvests, which may change the dynamics of nutritional deficiency symptomatology [2], although not assessed and described yet.

So, studying the deficiency symptoms and the nutritional status of the snap bean plants is crucial [3], especially in relation to macronutrients, which, at adequate concentrations, improve the visual, nutritional, and flavor quality of the pods. The identification of nutrient deficiency symptoms and associated it to a punctual nutrient deficit is complex due to the various biological functions and interactions that occur between nutrients and the environment [4]. Therefore, each plant species, when exposed to nutritional stress, exhibits different physiological mechanisms and nutrient absorption rate, which influence plant growth and development. The deficiencies caused by nutrients imbalance may reflect in lower photosynthetic efficiency, leaf area reduction, photosynthetic pigment content, among other alterations. These changes lead to a poor tissue formation, early senescence, and lower plant development, negatively influencing dry matter production [2] [5]. No studies on macronutrient omission in snap bean have been found, although this species has specific characteristics that can modify the physiological and nutritional mechanisms when subjected to nutritional deficiency.

In this context, it is necessary to know the dynamics of nutritional deficiency symptomatology and the physiological changes caused by macronutrient absence in snap bean, as well as seek answers to some questions. Which is the limiting macronutrient in the growth of snap bean? Are the effects of macronutrients on physiology and nutritional aspects variable for each nutrient and characteristic for the snap bean?

In this sense, this study aimed to assess macronutrient deficiency in physiological disturbances, growth, and characteristic visual symptoms of snap bean.

## Material and methods

### Growth conditions and plant material

**Fig 1:**
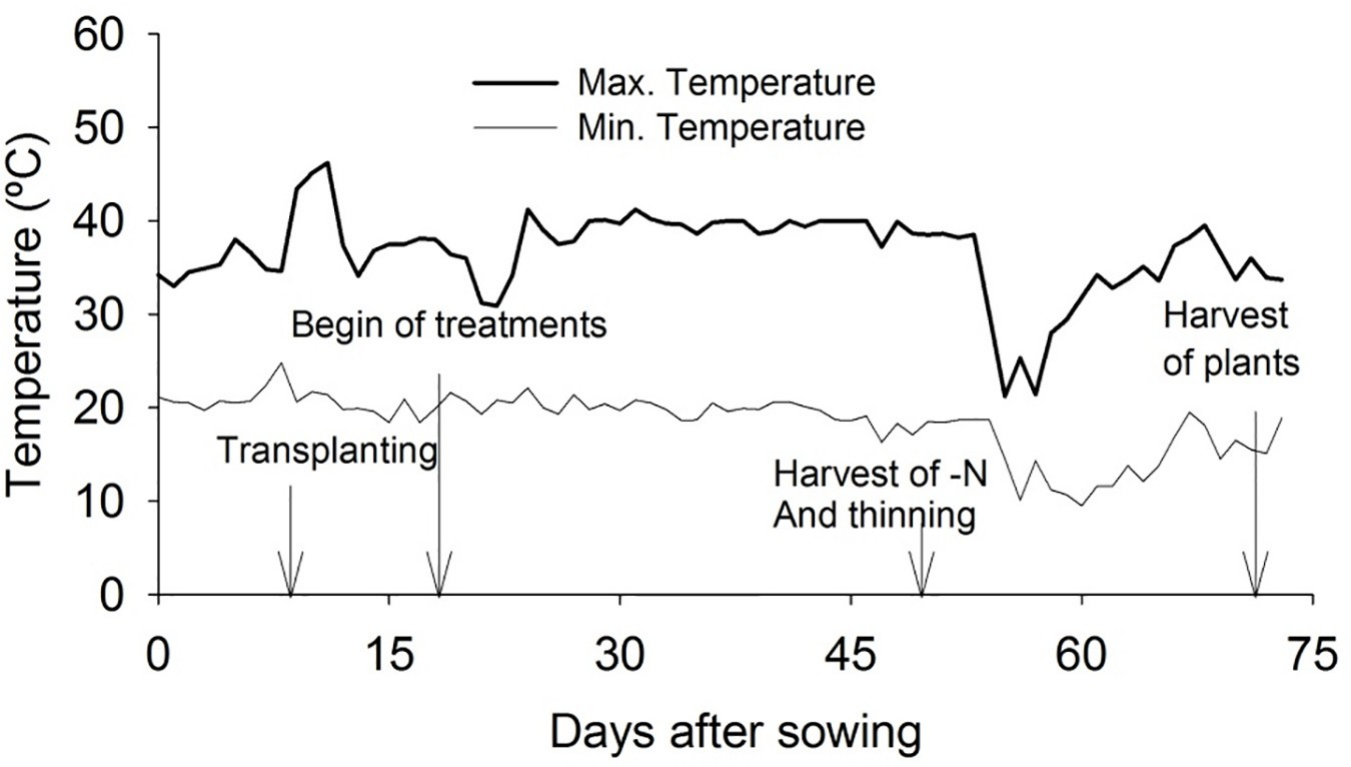
Maximum and minimum temperature (ºC) observed in the experiment.

The experiment was conducted in a greenhouse, at São Paulo State University (UNESP), Campus of Jaboticabal, SP, Brazil. The temperature data were collected during the experimental period. The brightness of the greenhouse was measured with a lux meter, with an average of 30% of the value found at full sun. Fig 1 contains the temperature variation during the experiment.

Snap bean seeds of the cultivar “Macarrão Trepador” were sown in polystyrene trays with 128 cells containing the commercial substrate Bioplant^®^-F class (producer). This substrate was made with raw materials from pine bark, coconut fiber, sphagnum peat and rice husk, with 55% of moisture, cation exchange capacity of 29.0 cmol_c_ dm^-3^, and pH 6.5. At 10 days after sowing (DAS), three seedlings were transplanted to each 5 dm^3^ polypropylene pot filled with vermiculite of fine granulometry. Vermiculite has uniformity in chemical and granulometric composition, porosity, water retention capacity of 50%, based on mineral clay type 2:1, cation exchange capacity of 5.0 cmol_c_ dm^-3^, and pH 6.5. Thinning was performed at 38 DAS, leaving only one plant per plot.

### Treatments and experimental design

The treatments consisted of providing a complete solution (CS) of Hoagland and Arnon [6] and others with the same solution but with suppression of macronutrients, with the absence of nitrogen (-N), phosphorus (-P), potassium (-K), calcium (-Ca), magnesium (-Mg) and sulfur (-S). The experimental design was a randomized block design with three replications.

Substrate moisture was maintained only with deionized water up to 12 DAS. From 13 to 15 DAS, a solution containing 0.75 mmol L^−1^ of ammonium nitrate was applied to avoid early N deficiency. At 16 DAS, treatments started to be applied with 25% dilution of the nutrient stock solution, which was increased to 50% at 19 DAS, and maintained at this level until the end of the experiment, being applied twice a day (in the morning and at the end of the day).

To avoid salinization, every 15 days, the substrate was washed with deionized water to avoid salinization, applying enough water to promote saturation and leaching of excess salts present in the substrate. The cultivation period of of plants in the macronutrients-free solution was maintained until the beginning of the severe deficiency symptoms, which occurred at 38 DAS for the N-free treatment, and at 71 DAS for the other treatments.

### Physiological analyses

Physiological analyses were carried out through the assessment of photosynthetic activity (A), stomatal conductance (Gs), and transpiration (E), using a Li-6400 (LICOR, USA). Water use efficiency (WUE) was calculated by the ratio of the net photosynthetic rate (A) to the transpiration rate (E).

The concentration of total chlorophyll (*Chl*T) and carotenoids was determined in the lower (LT) and upper (UT) third of leaves by extracting pigments with pure acetone [7]. The green color index (GCI) was measured on the first completely developed leaf (upper third—UT) and last completely developed leaf (the lower—LT), using the Opti-sciences^®^ CCM–200 chlorophyll meter.

### Vegetative growth and dry matter production

Leaf area was measured with a L-3100 (LICOR, USA). Root area was determined by the software Delta-TScan Root Analysis System.

At the end of the experiment, plants were separated into shoot and roots. Afterward, plant parts were washed in running water and then submerged in a neutral detergent solution (0.1% v/v) and hydrochloric acid solution (0.3% v/v) for approximately 20 seconds in each solution. Then, the leaves were washed with deionized water to remove the excess detergent and acid in the sample. The plant material was dried in a forced air circulation oven (65 ± 5°C) until reaching a constant mass. Shoot and root dry matter were obtained after drying.

### Visual analysis of symptoms

The plants were evaluated daily for the symptoms of nutritional deficiency, and representative images of symptoms were obtained when the severe deficiency symptoms showed up, at 38 DAS (N-free treatment) and 71 DAS (other treatments).

### Nutritional analyses of leaf tissue

The N content was determined by adding concentrated sulfuric acid to samples of previously dried and ground plant material, followed by distillation and titration with sulfuric acid [8]. The levels of P, K, Ca, Mg, and S were determined by the digestion of samples of plant material, using a digestive mixture of perchloric and nitric acid (1: 2), with readings of K, Ca, and Mg performed in spectrophotometry atomic absorption with air-acetylene flame and P and S readings through spectrophotometry [8]. Based on the content and shoot dry matter, the accumulation of each nutrient was determined in the plant shoot.

### Statistical analysis

The data were subject to analysis of variance by the F-test (p≤0.05 and p≤0.01). Subsequently, means were compared using the Scott-Knott test (p≤0.05). The analysis of the relationship between variables was carried out by the Pearson correlation (p≤0.05 and p≤0.01), using the statistical software AgroEstat^®^ version 2.0 [9].

A correlation network was used to graphically express the functional relationship between estimates of the Pearson’s correlation coefficients between variables, where the proximity between nodes (traces) was proportional to the absolute value of the correlation between those nodes. The thickness of the edges was controlled by applying a cut-off value of 0.60, which meant that only |r_ij_| ≥ 0.60 had their edges highlighted. Finally, positive correlations were highlighted in green, while negative correlations were represented in red.

## Results

**Fig 2:**
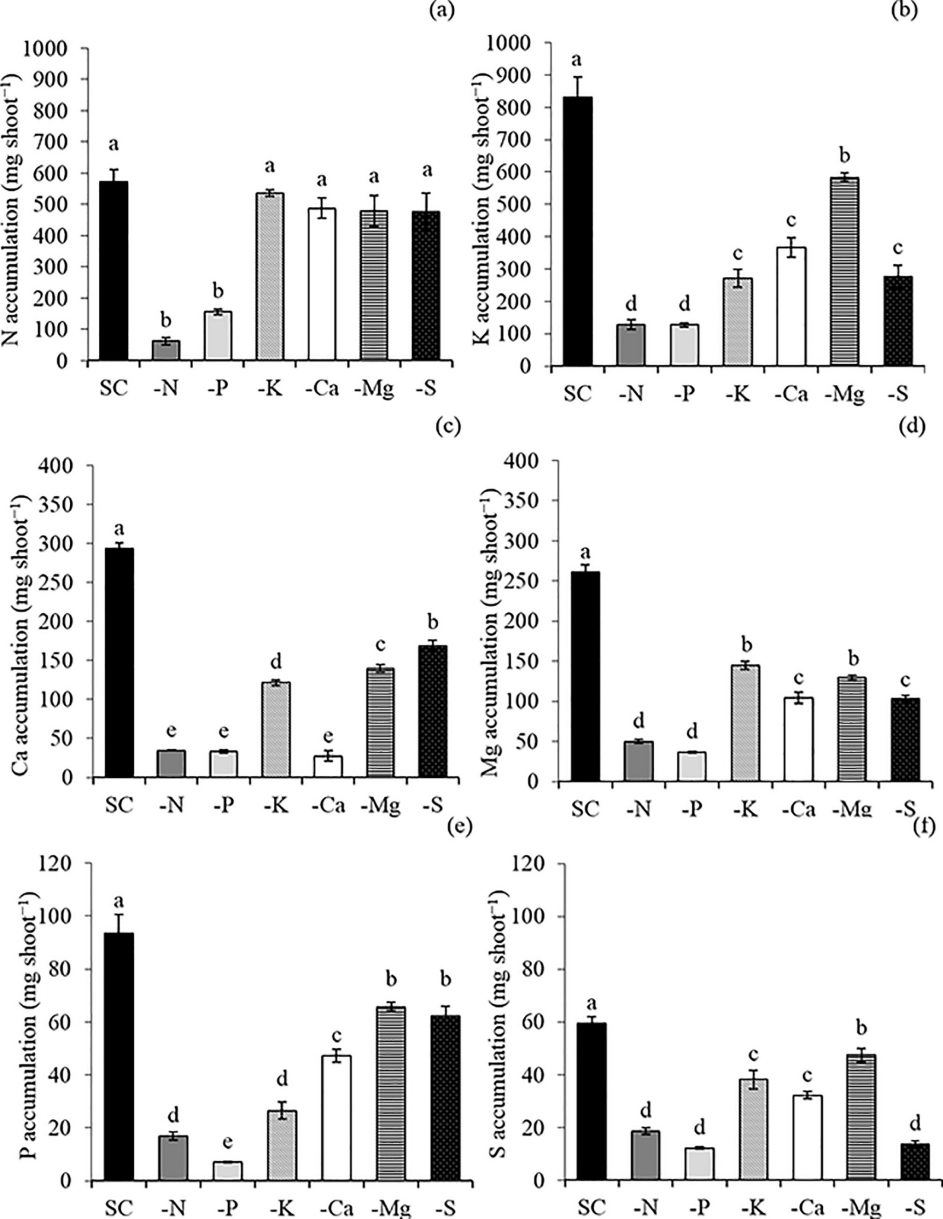
Accumulation (mg kg^-1^) of nitrogen (N) (a), potassium (K) (b), calcium (Ca) (c), magnesium (Mg) (d), phosphorus (P) (e), and sulfur (S) (f) in the shoot of snap bean in a complete solution (SC) of Hoagland and Arnon (1950), and with macronutrient-free solutions (−N, −P, −K, −Ca, −Mg, and −S). Means followed by the same letter in each bar did not differ from each other by the Scott-Knott test (p≤0.05). Bars represent the standard error of the mean.

The omission of macronutrients from the nutrient solution decreased the accumulation of nutrients in the shoot (Fig 2). This fact indicates the precision of this study in demonstrating less absorption of the plants due to the lack of nutrients in the nutrient solution. The macronutrient accumulation in the shoot of plants that received complete solution (SC) in relation to each omission (−) was 573/62 for SC_N_/−N, 93/7 for SC_P_/−P, 830/271 for SC_K_/−K, 293/27 for SC_Ca_/−Ca, 260/130 for SC_Mg_/−Mg and 59/14 for SC_S_/−S, respectively.

Each macronutrient-free solution has a different result regarding the nutritional balance and the physiological variables. For instance, nitrogen (N) omission decreased the accumulation of this element in the shoot (Fig 2A). In addition, plants grown in nutrient solution without N showed less accumulation of potassium (K) (Fig 2B), calcium (Ca) (Fig 2C), magnesium (Mg) (Fig 2D), phosphorus (P) (Fig 2E), and sulfur (S) (Fig 2F) when compared with plants grown in the complete nutrient solution (CS).

**Fig 3:**
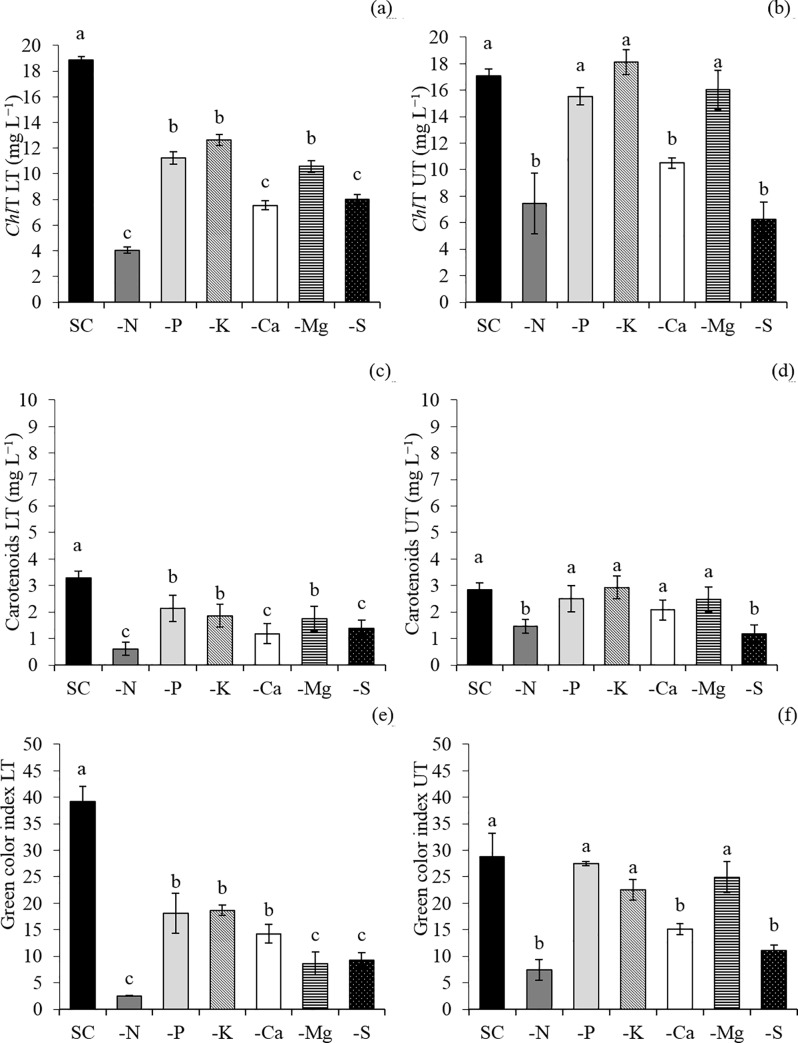
Total chlorophyll (*Chl*T) (a, b), carotenoids (c, d), and green color index (e, f) in the lower (LT) and upper (UT) third leaves of snap bean plants in a complete solution (CS) of Hoagland and Arnon (1950) and in micronutrient-free solution (−N, −P, −K, −Ca, −Mg, and −S). Means followed by the same letter in each bar did not differ from each other by the Scott-Knott test (p≤0.05). Bars represent the standard error of the mean.

**Fig 4:**
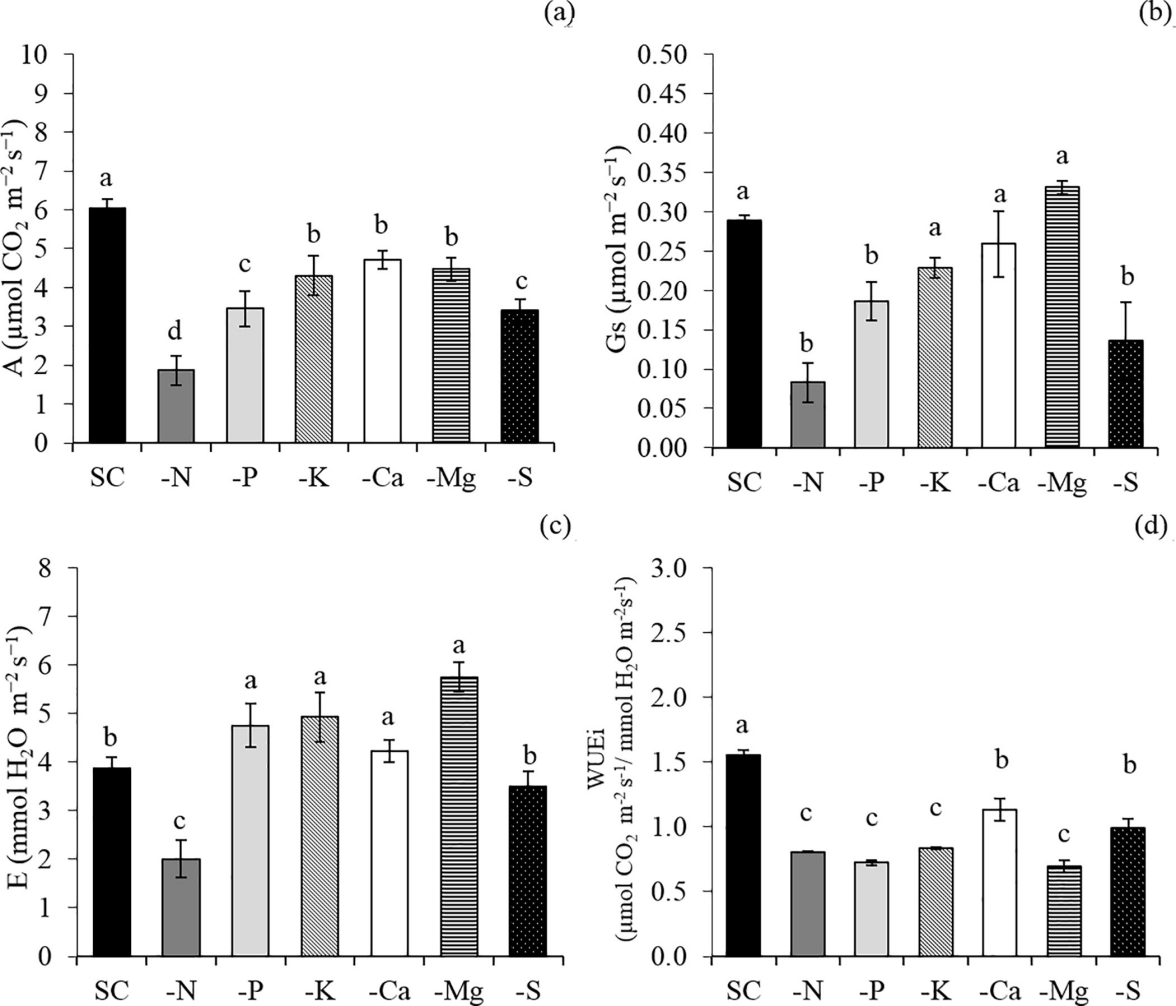
Photosynthesis rate (A) (a), stomatal conductance (Gs) (b), transpiration (E) (c), instantaneous water use efficiency (WUEi) (d) of snap bean plants in a complete solution (CS) of Hoagland and Arnon (1950) and in macronutrient-free solutions(−N, −P, −K, −Ca, −Mg, and −S). Means followed by the same letter in each bar did not differ from each other by the Scott-Knott test (p≤0.05). Bars represent the standard error of the mean.

**Fig 5:**
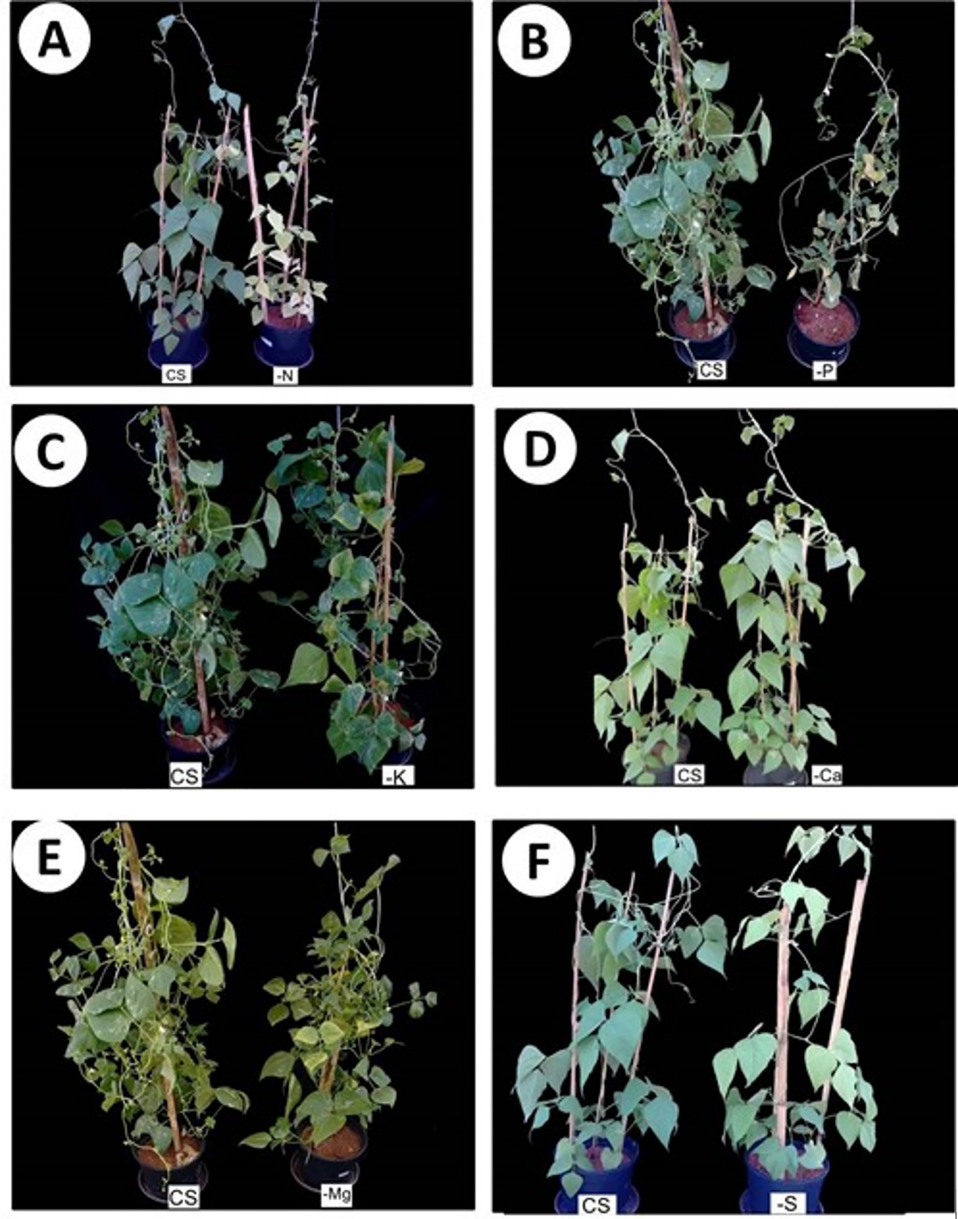
Visual symptoms of nutritional deficiency in leaves of snap bean plants cultivated in a complete solution (CS) of Hoagland and Arnon (1960) and with omissions (−) of nitrogen (a), phosphorus (b), potassium (c), calcium (d), magnesium (e) and sulfur (f).

**Fig 6:**
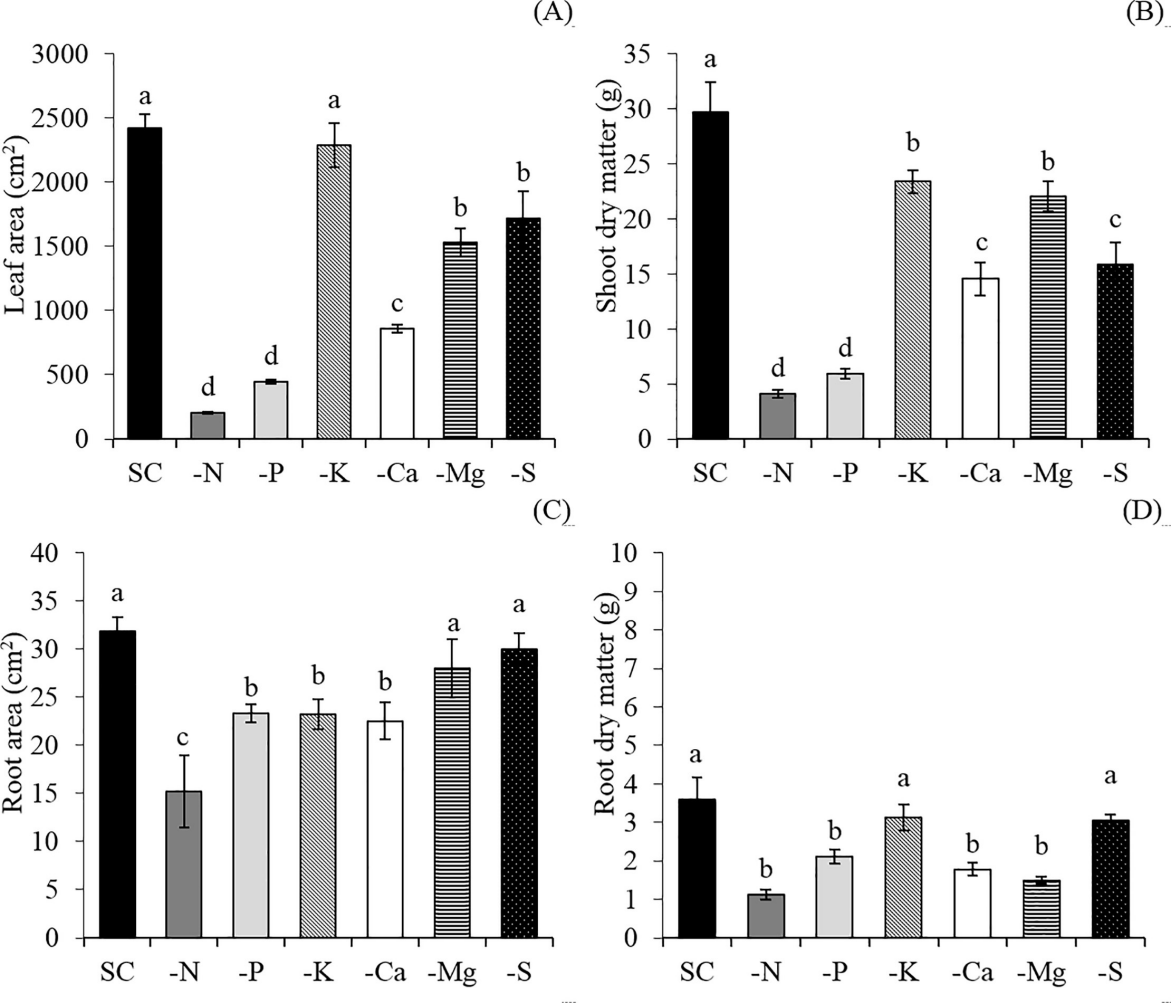
Leaf area (a), shoot dry matter (b), root area (c), and root dry matter (d) of snap bean plants in a complete solution (CS) of Hoagland and Arnon (1950) and in macronutrient-free solutions (−N, −P, −K, −Ca, −Mg, and −S). Means followed by the same letter in each bar did not differ from each other by the Scott-Knott test (p≤0.05). Bars represent the standard error of the mean.

The low macronutrients accumulation, caused by N suppression, affected plant growth since it took to a lower content of photosynthetic pigments (Fig 3A–3D) and green color index (Fig 3E and 3F), lower rates of photosynthesis (Fig 4A), stomatal conductance (Fig 4B) and transpiration (Fig 4C). The N deficiency also caused chlorosis of the lower- and middle- third leaves (Fig 3E and 3F) an necrosis of some lower-third leaves (Fig 5A), decreasing the leaf area (Fig 6A) and the dry matter accumulation (Fig 6B–6D), mainly in the shoot (Fig 6B).

K omission led to a lower content of K in the shoot (Fig 2B), also decreasing the accumulation of Ca (Fig 2C), Mg (Fig 2D), P (Fig 2E), and S (Fig 2F)when compared with the control treatment (CS). The photosynthetic rate (Fig 3A), is affected mainly due to the imbalance in the transpiration (Fig 4C), causing a lower water use (Fig 4D). The negative effects on the photosynthetic activity led to a decrease in the root area (Fig 6C) and the production of dry matter in the shoot (Fig 6B) and roots (Fig 6D). The symptoms of K deficiency in snap bean plants were characterized by the yellowing of leaf margins, starting with the older ones and progressing to the newly developed leaves (Fig 5C).

A Pearson’s correlation network was built to graphically verify the linear relationship between the main variables evaluated. Results revealed a high correlation between the variables used in the network. The contents of N, P, and Mg are correlated with each other and with shoot dry matter (SDM). In addition, P and Mg have a high correlation with instantaneous water use efficiency (WUE). Similarly, the contents of K, Mg, Ca, and S are highly correlated with each other in the central part of the graph, in addition to being correlated with photosynthesis (A), total chlorophyll, in the upper third of leaves (ChTU), and total chlorophyll, in the lower third of leaves (ChTL).

Plants cultivated in the absence of Ca had a low accumulation of Ca (Fig 2C), K (Fig 2B), Mg (Fig 2D), P (Fig 2E), and S (Fig 2F) when compared with the control treatment (CS), causing a decrease in the chlorophyll content (Fig 3A and 3B) and in the green color index (Fig 3E and 3F) of the lower- (LT) and upper- (UT) third of the plant, and in the carotenoids content of the lower-third of the plants (Fig 3C). This fact influenced the photosynthetic rate (Fig 4A), increased transpiration (Fig 4C), and decreased the water use efficiency (Fig 4D). All these changes sharply decreased the leaf area (Fig 6A) and root area (Fig 6C) and the shoot (Fig 6B) and root (Fig 6D) dry matter. Ca deficiency also decreased the size of new leaves with the curving of the edges downward (Fig 5D).

Mg omission led to lower content of Mg (Fig 2D), K (Fig 2B), Ca (Fig 2C), P (Fig 2E), and S (Fig 2F) in the shoot when compared with the control treatment (CS), causing a decrease in the content of chlorophyll (Fig 3A), carotenoids (Fig 3C), and green color index (Fig 3E) in the lower third of the plant, and in the photosynthetic rate (Fig 4A) and water use efficiency (Fig 4D) of the lower-third of the plant, leading to losses in leaf area (Fig 6A) and shoot (Fig 6B) and root (Fig 6D) dry matter. In addition, snap bean plants subjected to Mg deficiency showed internerval chlorosis lower- and middle-third leaves (Fig 5E).

P absence also decreased the P accumulation in the shoot (Fig 2E), associated with lower content of N (Fig 2A), K (Fig 2B), Ca (Fig 2C), Mg (Fig 2D), and S (Fig 2F)when compared with the control treatment (CS). Lower macronutrient content limited plant growth for it affected the contents of chlorophyll (Fig 3A), carotenoids (Fig 3C), and green color index (Fig 3E) in the lower-third leaves, as well as in decreased photosynthetic rate (Fig 4A), stomatal conductance (Fig 4B), increased the transpiration (Fig 4C) causing less the efficiency of water use (Fig 4D). The losses in photosynthetic activity caused by P deficiency sharply decreased the leaf area (Fig 6A) and the root area (Fig 6C), as well as the dry matter production of the shoot (Fig 6B) and roots (Fig 6D).

Plants cultivated in the absence of S had a low accumulation of S (Fig 2F), K (Fig 2B), Ca (Fig 2C), Mg (Fig 2D) and P (Fig 2E) in the shoot, causing a decrease in the content of chlorophyll (Fig 3A and 3B), carotenoids (Fig 3C and 3D), and in the green color index (Fig 3E and 3F) in the lower- and upper-third leaves. This fact affected the photosynthetic rate (Fig 4A), the stomatal conductance (Fig 4B), the water use efficiency (Fig 4D), the leaf area (Fig 6A), and shoot dry matter (Fig 6B) in relation to treatment with a complete solution (CS). The visual symptoms in the plants were evidenced by chlorosis in new leaves (Fig 5F).

Thus, the omissions of N and P in the nutrient solution led to lower accumulations of all macronutrients in the shoot. K, Ca, Mg, and S omissions decreased the amounts of K, Ca, Mg, P, and S in the shoot of the snap bean plants when compared with plants grown in the complete nutrient solution (Fig 2A–2F). Thus, from the losses in dry matter production of the shoot, the order of limitation of macronutrients in bean plants was N < P < Ca < S < Mg < K, with a decrease of up to 86.2%, 80.1%, 51.2%, 46.5%, 25.6%, and 19.3%, respectively (Fig 6A).

This result indicates that the deficiencies of N and P cause the greatest imbalances in the absorption of other macronutrients, with reflexes that decrease the production of dry matter by up to 80%. Then, the suppression of Ca and S limited the growth of plants with losses of up to 50%. And the omissions of Mg and K that affect less significantly the development of pod bean plants, however with decreases of about 20%. Therefore, there was a strong influence of nutritional balance, on growth and in dry matter production.

## Discussion

In snap beans plants, the omissions of nitrogen (N) and phosphorus (P) in the nutrient solution led to lower accumulations of all macronutrients in the shoot. This fact indicates that the deficiencies of N and P cause a strong imbalance in the absorption of potassium (K), calcium (Ca), magnesium (Mg), and sulfur (S). This is due to the biological structural role of N in the constitution of enzymes and transporters involved in the absorption of nutrients and P in the composition of ATP, which is used during the process [2].

Due to nutritional waste, the various biological functions and interactions that occur between nutrients and the environment have been modified, causing disturbances in the physiological mechanisms of plants [4]. The concentration of N in the leaf directly interfered with the pigments as it is a structural nutrient of the chlorophyll molecule [10]. This decrease reflected on a decrease in the photosynthetic rate because photosynthetic pigments play a key role in transferring excitation energy to photosystems [11].

Allied to this, the plants showed a decrease in water use efficiency (WUE), i.e., a higher water consumption per unit of fixed carbon [12]. This fact can be attributed to the lesser activity of photosynthetic enzymes [13], caused by the lower P accumulation in the leaf tissue. P is linked to metabolic activities, and its low supply directly affects gas exchange, photosynthetic rate, and the activity of ribulose 1,5-bisphosphate carboxylase (rubisco) [14].

P omission also causes hormonal imbalance in the root, reducing auxin concentration and increasing the concentration of cytokinins and abscisic acid. The result is the decrease in lateral root formation [15], causing a reduction of the root area and dry matter (Fig 6C and 6D), a result of the lower branching capacity and lateral root formation. The deficiency of P caused purplish tones only in the lower third leaves, following a common symptomatology gradient for the deficiency of nutrients of a high mobility in the plant (Fig 5B).

**Fig 7:**
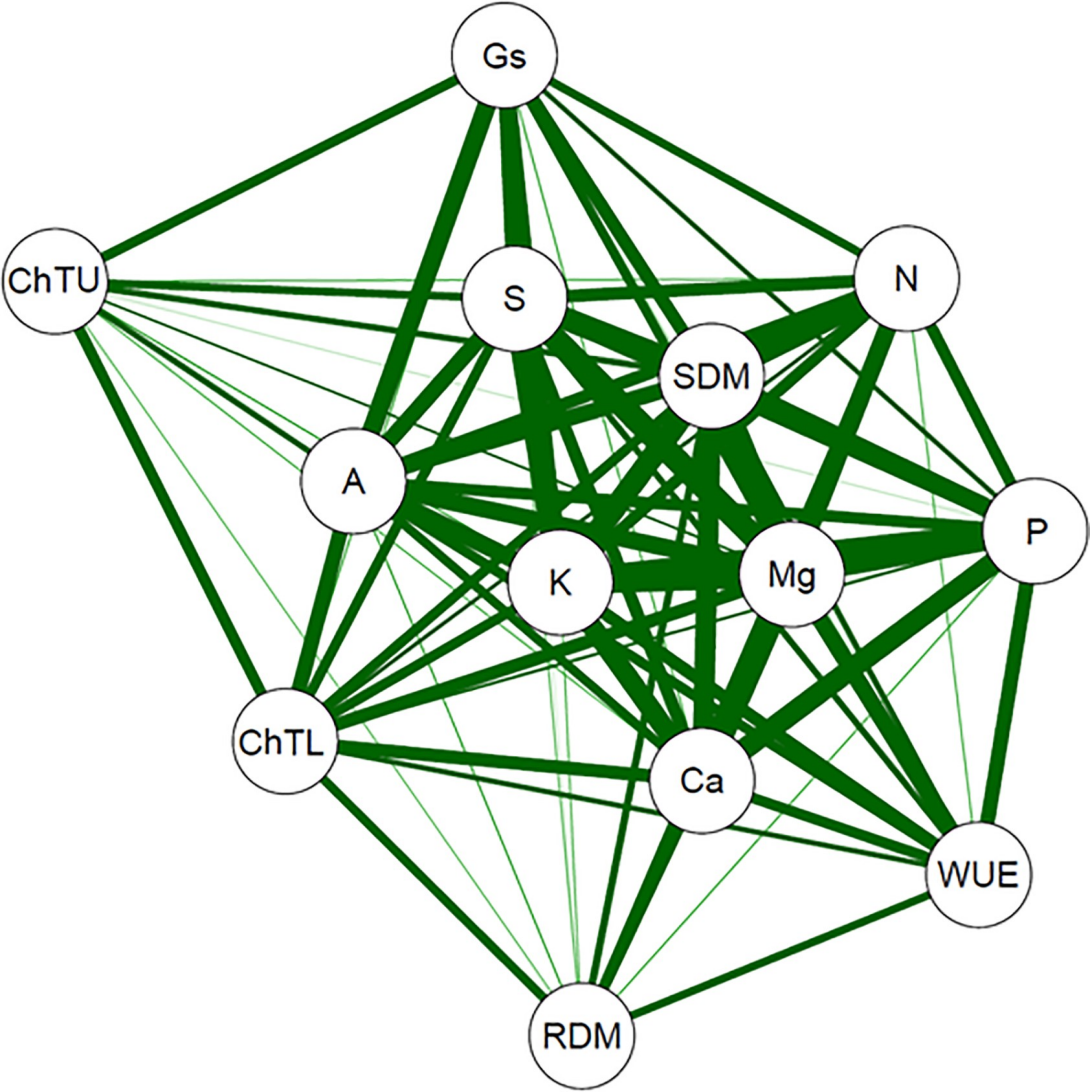
Pearson’s correlation network between nutritional and physiological variables evaluated in snap bean plants. N: nitrogen accumulation; P: phosphorus accumulation; K: potassium accumulation; Ca: calcium accumulation; Mg: magnesium accumulation; S: sulfur accumulation; ChTU: total chlorophyll in the upper third of leaves; ChTL: total chlorophyll in the lower third of leaves; A: photosynthesis; Gs: stomatal conductance; WUE: instantaneous water use efficiency; RDM: root dry matter; SDM: shoot dry matter.

Since the P present in the cell is used primarily in the metabolism linked to the plant energy system, being constituent of NADP and ATP [16] and in phospholipid formation, which are molecules constituting the membrane cell, a deficiency condition of P cause decrease in phospholipid formation, leading to a lower formation of new cells and cell elongation [17]. And when P deficiency is associated with low N content (Fig 7), as observed in N and P omissions, the smallest number of soluble proteins, accentuating the limitation the formation of new tissues in the plant [18]. For these reasons, N was the most limiting nutrient for the growth of snap bean plants, decreasing up to 86.2% the shoot dry matter production, followed by P omission, with decreases of up to 80.1% in the shoot dry matter production. Finally, Ca was the third macronutrient that most limited plant growth, causing a decrease of up to 51.2%.

The lowest plant development observed in Ca omission was caused mainly by decreasing the amount of photosynthetic pigments that affected the shoot development. This fact was due to a lower expansion and lack of rigidity of the cell wall, which is governed by Ca^2+^ bonds to pectates [19]. Thus, Ca deficiency reduced the size of new leaves, with curving of the margins downward (Fig 5D) due to cell wall malformation. In addition, Ca within the cytosol can also act as a signal for several enzymes linked to photosynthesis and secondary messenger in stresses of both biotic and abiotic nature [19], contributing to the observed decrease in the photosynthetic rate (Fig 4A) and dry matter accumulation (Fig 6B), up to 51.2% in the shoot.

S deficiency caused a decrease in the amount of photosynthetic pigments, and this occurred because the chloroplasts tilacoid membranes are composed of sulfolipid compounds [20]. Thus, S deficiency inhibits the synthesis of thylacoidal membranes and promotes chlorophyll deficiency. On top of that, the low S content possibly caused a degradation of the Rubisco enzyme, indicating a link between carbon and S metabolism [20]. The nutritional deficiency of S is also commonly related to a great decline in protein formation because it is the constituent of amino acids, such as cysteine and methionine [21]. These factors combined caused an imbalance in the photosynthetic rate and protein production, leading to lower growth and losses in dry matter accumulation of up to 46.5% (Fig 6).

Mg was the fifth most limiting macronutrient for the shoot development of snap bean plants. Mg is a basic constituent of chlorophyll molecules, and about 10–20% of the Mg present in the plant is bound to these pigments. In addition, low amounts of this element can promote a breakdown of chlorophyll molecules in older leaves, being translocated to the younger tissues, explaining the lower *Chl*T concentration in the lower parts of plants [22]. A continuous deficiency leads to a lower sugar transport inside the phloem since Mg acts as a carrier of these compounds, in addition to causing a decrease in the synthesis of ribosomes and acceleration in root senescence. This process negatively affects root development, which can justify a decrease in root dry matter in relation to the control treatment (complete solution) [23].

In addition, chlorophyll decomposition under Mg deficiency may be related to the accumulation of sugars and starch in the cells of deficient leaves. This fact causes a super-reduction of the transport chain of photosynthetic electrons, which leads to the formation of reactive oxygen species and, consequently, to chlorophyll destruction [24] and intensification of visual symptoms.

K omission affected the photosynthetic rate mainly due to the imbalance in the transpiration since K is strongly linked to the regulation of the stomatal opening, loading of photoassimilates in the phloem, maintenance of membrane potential, and activation of many enzymes related to the photosynthesis metabolism [25]. Thus, the loss of dry matter observed in the treatment with K omission may have been more strongly influenced by the performance of the nutrient in the processes of enzymatic activation when compared to the control treatment (complete solution). The deficiency of K caused chlorosis on the leaf margins, being more evident in the middle third of the plants (Fig 5C). However, leaf area and shoot dry matter decreased less under the omission of this nutrient (Fig 6A and 6B), probably because the role of K^+^ in the osmotic function can be partially replaced by other cations such as Mg^2+^, Na^+^ or Ca^2+^ [26]. This may have occurred in the snap bean growth, considering that Mg and Ca accumulation was quite expressive in the treatment −K (Fig 2C and 2D). This result suggests that these might be the reasons why K was the nutrient that least influenced shoot growth.

Even under conditions of macronutrient omission, the global effect of the nutritional balance was observed, which was demonstrated by the positive correlation between nutrient accumulations (Fig 7). This characteristic may justify the lesser expressive effect for some nutrients. Except for *Chl*T in the upper third and WUE, N accumulation revealed a positive correlation with all other variables (Fig 7). The imbalance caused by the deficiency of this nutrient reduced the photosynthetic efficiency, the content of photosynthetic pigments of the lower third, sugar translocation, besides causing other alterations. These changes triggered tissue malformation, early senescence of tissues, and lower plant development, leading to negative reflexes in plant growth.

Some studies have reported that the omission of N, P, K, and Ca strongly influences, negatively, the growth of common beans plants at the vegetative stage, unlike Mg and S [27]. Results show that the order of demand for macronutrients did not coincide with the nutritional limitation observed in the growth of common beans, in which the most limiting macronutrients were N, P, Ca, S, Mg, and K. This fact justifies the higher concern regarding nutritional management with Ca and S in snap beans and the need for more research on this crop.

## Conclusions

Macronutrient accumulation in the shoot of plants that received the complete solution (CS) in relation to each omission was 573/62, 93/7, 830/271, 293/27, 260/130, and 59/14 for CS_N_/−N, CS_P_/−P, CS_K_/−K, CS_Ca_/−Ca, CS_Mg_/−Mg, and CS_S_/−S, respectively.

With the lowest macronutrients accumulation, the content of photosynthetic pigments and the photosynthetic rate were reduced in bean plants, leading to harmful effects on plant growth. Thus, from the losses in shoot dry matter production, the order of limitation of macronutrients in bean plants was N < P < Ca < S < Mg < K, with a decrease of up to 86.2%, 80.1%, 51.2%, 46.5%, 25.6%, and 19.3%, respectively.

Nitrogen deficiency is more evident, with appearance of chlorosis in the leaves of the lower- and upper-third leaves and necrosis in the lower-third leaves.

## Supporting information

S1 Table Treatment averages for the evaluated variables. N: nitrogen accumulation; P: phosphorus accumulation; K: potassium accumulation; Ca: calcium accumulation; Mg: magnesium accumulation; S: sulfur accumulation; ChTU: total chlorophyll in the upper third of leaves; ChTL: total chlorophyll in the lower third of leaves; A: photosynthesis; Gs: stomatal conductance; WUE: instantaneous water use efficiency; RDM: root dry matter; SDM: shoot dry matter.
(XLSX)
